# Transcriptome profiling of the salt-stress response in *Triticum aestivum* cv. Kharchia Local

**DOI:** 10.1038/srep27752

**Published:** 2016-06-13

**Authors:** Etika Goyal, Singh K. Amit, Ravi S. Singh, Ajay K. Mahato, Suresh Chand, Kumar Kanika

**Affiliations:** 1 Banasthali University, Banasthali, Rajasthan, India; 2 Biotechnology and Climate Change Laboratory, ICAR-NRC on Plant Biotechnology, New Delhi, India; 3 Devi Ahilya University, Indore, India

## Abstract

Kharchia Local wheat variety is an Indian salt tolerant land race known for its tolerance to salinity. However, there is a lack of detailed information regarding molecular mechanism imparting tolerance to high salinity in this bread wheat. In the present study, differential root transcriptome analysis identifying salt stress responsive gene networks and functional annotation under salt stress in Kharchia Local was performed. A total of 453,882 reads were obtained after quality filtering, using Roche 454-GS FLX Titanium sequencing technology. From these reads 22,241 ESTs were generated out of which, 17,911 unigenes were obtained. A total of 14,898 unigenes were annotated against nr protein database. Seventy seven transcription factors families in 826 unigenes and 11,002 SSRs in 6,939 unigenes were identified. Kyoto Encyclopedia of Genes and Genomes database identified 310 metabolic pathways. The expression pattern of few selected genes was compared during the time course of salt stress treatment between salt-tolerant (Kharchia Local) and susceptible (HD2687). The transcriptome data is the first report, which offers an insight into the mechanisms and genes involved in salt tolerance. This information can be used to improve salt tolerance in elite wheat cultivars and to develop tolerant germplasm for other cereal crops.

Salinity stress is a major factor limiting the productivity of crop plants and majority of the crop plants are sensitive to salinity caused by high concentrations of salts in the soil. More than 20% of total irrigated land has been damaged by salt worldwide and nearly 1.5 Million hectare (M ha) is being rendered useless each year due to high salinity^1^,^2^. This increasing stalinization of agricultural land has global effects, resulting in losses of up to 50% cultivable land by 2050 century^3^. In most of the cases, the deleterious effects of salinity have been associated with increase in Na^+^ and Cl^−^ ions causing many physiological disorders in plants^4^. Salinity at physiologically toxic level induces membrane damage, nutrient unavailability, altered levels of growth regulators, enzymatic inhibition and metabolic dysfunction including photosynthesis which ultimately leads to death of the plant^4^,^5^,^6^. Therefore, the efforts to increase salt tolerance of crop plants are of immense importance for sustainable agriculture and could also potentially improve crop yield^5^.

Plants employ various mechanisms to deal with salt stress; the most common one involved minimization of the amount of salt taken up by roots and its partitioning at tissue and cellular levels to avoid buildup of toxic concentrations in the cytosol of functional leaves. Plants have special mechanisms including ion homeostasis, osmotic homeostasis, redox equilibrium, growth regulation and others to combat salt stress. These mechanisms are achieved through corresponding physiological and biochemical changes facilitated by the expression of numerous salt-responsive genes. These include group of structural protein-coding genes, including osmoregulatory genes, antioxidant proteins, late embryogenesis abundant (LEA) proteins, transporters/antiporters, transcription factors (TFs), such as ERF and WRKY and signal-related protein kinases^6^. The interaction among the above mentioned genes forms basis of several pathways such as the salt overly sensitive (SOS) pathway, the calcium-dependent protein kinase (CDPK) pathway and the mitogen-activated protein kinase (MAPK) pathway^7^,^8^,^9^,^10^. Furthermore, plant hormones, such as abscisic acid (ABA), ethylene, salicylic acid and jasmonic acid, also play important roles in stress signaling and adaptation^11^,^12^,^13^. In spite of the progress made in elaborating these processes, the complexity of interactions involved in the salt stress tolerance mechanisms in plants necessitates more indepth analysis. Hence, there is a need to develop a clear understanding of salt stress tolerance mechanism of plants and identify the mechanism involved in imparting salt tolerance.

It is well known that hexaploid bread wheat shows higher salt tolerance than its tetraploid progenitor, *Triticum turgidum*^14^. The higher ploidy level and complex genome composition of hexaploid wheat, provides it greater physiological and ecological plasticity than its tetraploid and diploid progenitors^15^. The salt-tolerant trait in wheat cultivars grown in India, has been derived from land race Kharchia local, which is a selection from farmer’s field of salinity affected Kharchi-Pali area of Rajasthan^16^. Therefore, the salt tolerant Kharchia local landrace is an excellent genetic resource for deciphering mechanism of salt tolerance in wheat (*Triticum aestivum*).

RNA-sequencing techniques have proven to be advantageous and economical for transcriptomic studies both in model and non-model plant species, as they neither require previously annotated genome, nor pre-synthesized nucleotide as probes. These techniques are also not limited by Expressed Sequence Tag (EST) availability^17^. The 454-FLX massively parallel DNA sequencing platform is a widely used next generation sequencing technology. It has enabled studies of the global transcriptome of even non-model plant species^18^. Large number of studies on the high throughput sequencing of plant transcriptome in many model and non model species, such us maize, grapevine, *Taxus*, eucalyptus, olive and cucumber etc. have been undertaken^19^,^20^,^21^,^22^,^23^. Comparative global transcriptome analysis is a powerful approach for discovering the molecular basis underlying specific biological events^24^,^25^,^26^. This approach not only allows us to map change in gene expression under specific conditions, but also to detects unique transcripts^27^,^28^,^29^.

In the present investigation, we studied the expression patterns of genes involved in salt tolerance of Kharchia Local. A comparison of the expression pattern of some of the selected genes was carried out in Kharchia Local (salt tolerant) and HD2687 (susceptible) cultivar of wheat. To our knowledge, this is the first attempt to analyse *T. aestivum* cv. Kharchia Local root transcriptome under salt stress. The data generated from this study, will serve as a blueprint of gene expression profile in roots of wheat under salt stress. Comparative root transcriptome profiling under salt stress, will also help to unravel the network of differentially expressed genes in the salt-tolerant wheat cultivar.

## Results

### Physiological and Biochemical Analysis

Physiological and biochemical analysis i.e. Relative Water content (RWC), Membrane Stability Index (MSI), Chlorophyll content (CHL), Carotenoid (CAR) and Proline content were carried out in control and salt stressed seedlings of wheat cv. Kharchia local. We observed decrease in RWC, MSI, CHL and CAR content under salt stress (250 mM NaCl) in comparison to control samples (Supplementary Fig. S1a–d). An increase in proline and total soluble sugars content under salt stress was observed (Supplementary Fig. S1e,f).

### Sequencing and *de novo* transcriptome assembly of *T. aestivum*

In order to study gene expression pattern in response to salt stress, total RNA was extracted from root samples of *T. aestivum* control (CWR) and salt treated (TWR) seedlings. There were three biological replicates and three technical replicates of each, from which equimolar amount of RNA was pooled for cDNA synthesis. High throughput sequencing was performed using Roche 454 GS FLX Titanium sequencing platform. This sequencing run produced a total of 106,649 and 347,233 raw reads in CWR and TWR samples, respectively. After removal of low quality, adapter and barcode sequences a total of 367,022 (CWR- 98,245; TWR- 268,777) high quality reads totaling 222,600,000 bp were obtained. An overview of the sequencing and assembly process is presented in Table 1. *De novo* assembly of these high quality cleaned reads generated 22,531 ESTs. Using CD-HIT (V.4.6.1) software^30^, the ESTs were further assembled into 17,911 unigenes, with a minimum unigene size of 201 bp, a maximum size of 3,602 bp and an average length of 580 bp. The size distribution of the unigenes is shown in Supplementary Fig. S2a. Among these unigenes, 8,573 (47.86%) were longer than 500 bp, out of which 1,950 (22.75%) were greater than 1,000 bp. GC content gives important indication about the genes and genomic composition including evolution, structure (intron size and number) and gene regulation. It is also an indicator of stability of DNA. The average GC content of *T. aestivum* unigenes was 51.4% (Supplementary Fig. S2b), which is in range of GC levels of coding sequences in monocots^31^. The transcriptome dataset generated (raw data) from the present study has been submitted at the Sequence Read Achieve (SRA), National Centre for Biotechnology Information (NCBI) with the accession numbers SRR1300785 and SRR1300786 for CWR and TWR, respectively.

### Functional annotation and classification of *T. aestivum* transcriptome sequences

**Figure 1:**
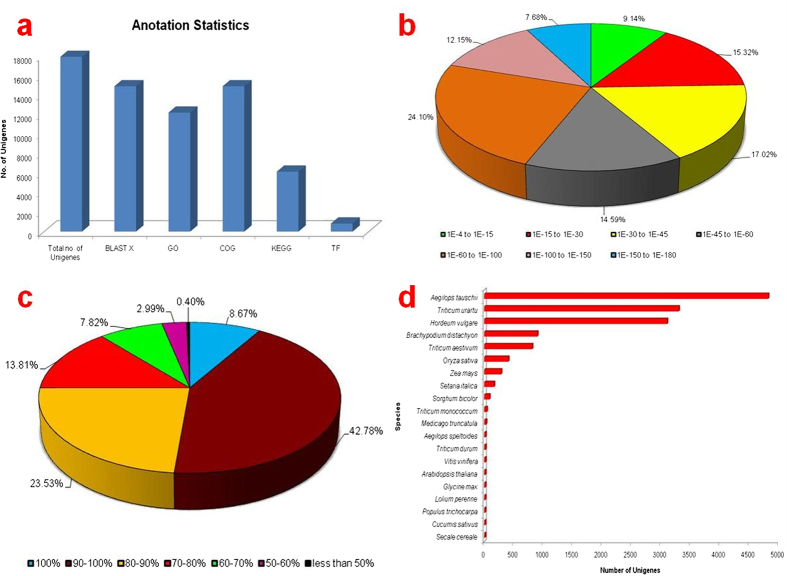
Homology search of *T. aestivum* under salt stress. (**a**) Annotation statistics of *T. aestivum* transcriptome; (**b**) E-value distribution of the BLASTX hits against the nr protein database for each unigene, using an E-value cutoff of 1.0 E^−3^; (**c**) Similarity distribution of the top BLAST hits for each unigene; (**d**) Species distribution of sequences.

Functional annotation of assembled sequences was compared against the NCBI non redundant (nr) protein database with a cut-off E-value of 1.0 E^−3^ using Blast2GO^32^,^33^,^34^. Of the, 17,911 assembled unigenes, 14,898 (83.18%) aligned to nr protein database while the remaining 3,013 (16.82%) did not show homology to any sequence in the database (Supplementary Table S1, Fig. 1a). Among the aligned unigenes, 54.32% had an E-value of less than 1.0 E^−50^ and showed very strong homology with the gene sequence in the database. The remaining 45.68% unigenes had an E-value ranging from 1.0 E^−4^ to 1.0 E^−50^ (Fig. 1b). The similarity distribution showed that 51.45% of the aligned unigenes had homology more than 90% whereas, 48.15% showed homology between 50 to 90% and only 0.40% had lower than 50% (Fig. 1c). To study the sequence conservation of *T. aestivum* compared to other plant species, we analyzed the species distribution of the unigene dataset by aligning sequences against the nr protein database. Approximately 89.64% of total unigenes matched with sequences from six top-hit species, i.e., *Aegilops tauschii* (32.67%), *Triticum urartu* (22.16%), *Hordeum vulgare* (20.84%), *Brachypodium distachyon* (6.06%), *Triticum aestivum* (5.47%) and *Oryza sativa* (2.75%) all of which are members of family *Poaceae*. The twenty top-hit species based on nr annotation are shown in Fig. 1d.

**Figure 2:**
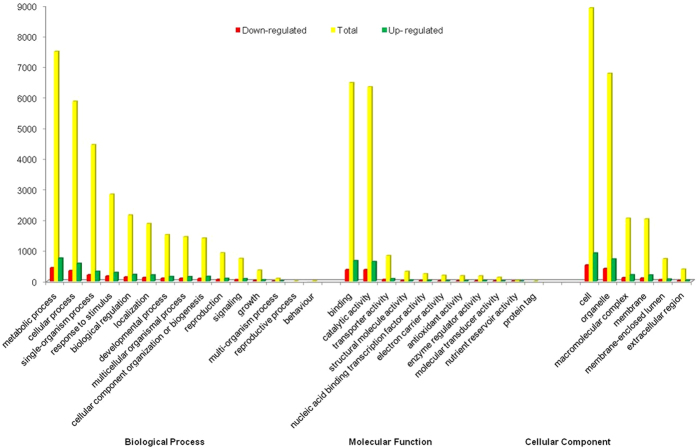
GO classification of *T. aestivum* transcriptome and differentially expressed genes between control and salt stress. The Y-axis represents number of unigenes and X-axis shows the GO categories.

Sequence homology based on GO classification using Blast2GO tool revealed that out of all the assembled unigenes 12,199 were summarized under three main GO categories, including 32 functional groups (Fig. 1a). A total of 71,516 GO assignments were obtained, of these, 48.66% comprised the largest category - biological processes, followed by cellular component (26.95%) and molecular functions (24.39%) at level 2. In “biological process” category, the majority of unigenes were involved in “metabolic process” (24.04%), “cellular process” (18.82%) and “single-organism process” (14.28%). With respect to “cellular component”, “cell” (42.67%), “organelle” (32.44%) and “macromolecular complex” (9.83%) were the dominant groups. Under “molecular function”, the top three categories were “binding” (43.51%), “catalytic activity” (42.60%) and “transporter activity” (5.60%) (Supplementary Table S2, Fig. 2).

**Figure 3:**
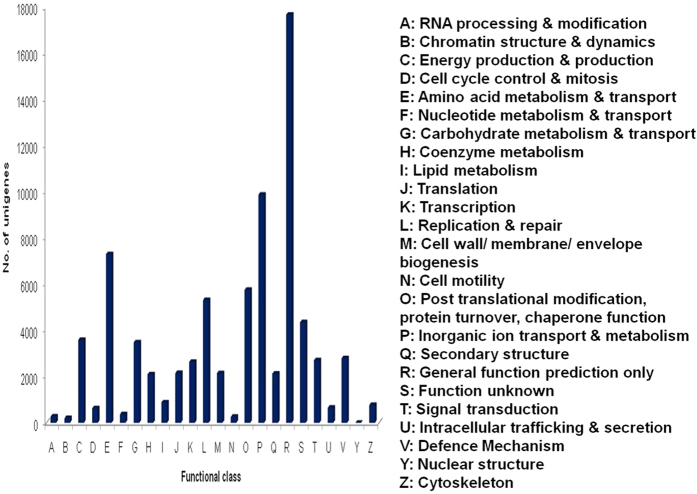
COG function classification of *T. aestivum* transcriptome. The number of unigenes is reported on Y-axis.

COG classification of 14,898 nr hits indicated that 9,305 (62.46%) unigenes clustered into 24 functional categories (Fig. 1a). Some unigenes had multiple COG functions resulting in, 78,102 functional annotations. Among the 24 functional categories, “general functional prediction only” (17,669, 22.62%) represented the largest group, followed by “inorganic ion transport and secretion” (9,881, 12.65%), amino acid metabolism and transport” (7303, 9.35%), “post translational modification, protein turnover, chaperone function” (5761, 7.38%), “replication and repair” (5318, 6.81%), “function unknown” (4356, 5.58%), “energy production and conversion” (3596, 4.60%), “carbohydrate metabolism and transport” (3486, 4.46%), “defense mechanism” (2799, 3.58%) and “signal transduction” (2705, 3.46%). The smallest groups belonged to “cell motility” (261, 0.33%), “chromatin structure and dynamics” (202, 0.26%) and “nuclear structure” (5, 0.006%) (Fig. 3).

For a better understanding of the interactions of the putative proteins and biological functions obtained from 17,911 unigenes, a single-directional BLAST search against KEGG protein database^35^ was performed. This is an alternative approach to categorize genes functions with emphasis on the biochemical pathways. A total of 6,143 unigenes had KEGG orthologous number in our study, which in turn were involved in 310 different pathways (Fig. 1a). Summary of the sequences involved is described in Supplementary Table S3. The five largest pathway groups were “ribosome” (ko03010), “spliceosome” (ko3040), “carbon metabolism” (ko01200), “oxidative phosphorylation” (ko00190) and “RNA transport” (ko03013).

The 6,143 KEGG annotated unigenes were categorized into six different functional groups (Table 2). Of these, 1,390 unigenes, were classified into the “metabolism”, with most of them involved in “carbohydrate metabolism” (18.27%), “amino acid metabolism” (16.55%), “energy metabolism” (10.93%), “lipid metabolism” (9.42%), “biosynthesis of other secondary metabolites” (4.39%) and other sub-categories. This suggests that carbohydrate, amino acids and energy metabolism were active under salt stress. Unigenes encoding core component in the plant hormone signal transduction pathways, including “ABA” (abscisic acid), “BR” (brassinosteroid) and “ethylene signaling pathways” were also prominently present. These pathways were well documented to play role in acclimatization of plant under stress conditions^36^. In the secondary metabolism categories, the most represented subcategories were “phenylpropanoid biosynthesis” (46.72%), “flavonoid biosynthesis” (9.49%), “tropane, piperidine and pyridine alkaloid biosynthesis” (8.76%) and many more. In addition to metabolism, sequences were also classified into the “genetic information processing (GIP)”, which accounted for 633 unigenes of the KEGG annotated sequences, most of them were involved in “translation” (39.97%), followed by “folding, sorting and degradation” (31.12%). “Cellular processes” were represented by 289 unigenes consisting of “transport and catabolism”, “cell growth and death”, “cell motility” and “cell communication”. Additionally, 218 unigenes were classified into “environmental information processing (EIP)” including “signal transduction”, “signaling and interaction molecules” and “membrane transport”.

### Identification of Transcription Factors

Transcription factors are important upstream regulatory protein which plays a crucial role in various developmental processes and in responses to abiotic and biotic stresses. In the present study, we identified a total of 826 unigenes, representing 4.61% of the transcriptome classified into 77 putative transcription factors (TF) families (Supplementary Table S4). Among the 77 TF families, “WD40-like” represented the most abundant category comprising of 139 (16.82%) unigenes, followed by 107 (12.95%) unigenes representing “C2H2” and 39 (4.72%) unigenes representing “MYB-HB-like” transcription factor.

### Quantification of *T. aestivum* transcripts and analysis of Differentially Expressed Genes (DEGs) in *T. aestivum* transcriptome

**Figure 4:**
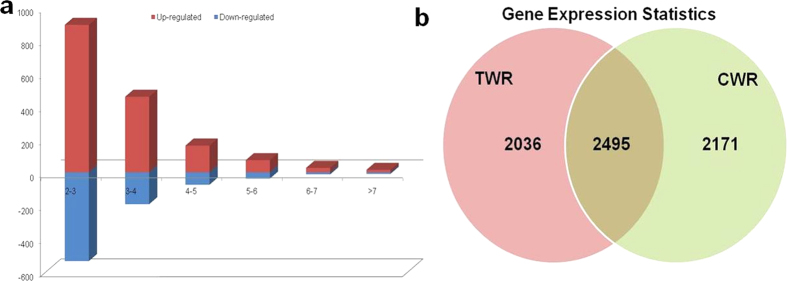
Overview of differentially expressed genes between control and salt stress in *T. aestivum*. (**a**) Fold change distribution of differentially expressed genes; (**b**) Out of 17,911 unigenes, 2,495 were differentially expressed, including 865 up-regulated and 1,630 unigenes as down-regulated. 2,171 unigenes were uniquely expressed in control, whereas, 2,036 unigenes were uniquely expressed under salt stress.

To compare the expression level of unigenes in the CWR and TWR libraries, the number of clean reads were compared with assembled unigenes. Mapping of all the reads onto the non-redundant set of *T. aestivum* unigenes revealed that the number of reads corresponding to each unigene ranged from 3.2 to 323.73 for CWR (Reads Per Kilobase of Exon Per Million Fragments Mapped) and from 4.74 to 224.675 for TWR library, respectively, indicating a very wide range of expression levels of wheat unigenes. It also indicated that very low expressed unigenes were also represented in the present assembly. Among all the assembled unigenes, 4,531 unigenes were in TWR. Out of these 2,036 unigenes were unique in salt treated seedlings. Out of 4,666 unigenes in CWR, 2,171 unigenes were unique in control samples, illustrating that 2,495 unigenes were expressed both in control and salt treated plants. (Supplementary Table S5; Fig. 4a,b).

#### Gene ontology analysis

To study the functions of DEGs, GO terms were extracted using Blast2GO tool and subjected to GO enrichment analysis. Annotation of DEGs revealed 1,977 unigenes belonged to 30 GO groups while the remaining 518 unigenes were not classified (Fig. 2; Supplementary Table S2). GO functional enrichment analysis of differentially expressed genes revealed that they were involved in “iron ion binding”, “oxidoreductase activity”, “hydrolase activity”, “ion binding”, “cation binding”, “cell growth” and “pyrophosphate activity”. Most of the unigenes up-regulated in TWR were involved in “oxidoreductase activity”, “heme binding”, “iron ion binding”, “anatomical structural morphogenesis”, “ATPase activity”, “single organism process” and “transition metal ion binding”. This suggested that these functions were more active under salt stress. In contrast, the GO groups “microtubule-based process”, “chromatin binding”, “motor activity” and “cytoskeleton protein binding” were over-represented in the up-regulated unigenes of the CWR (Table 3).

### Validation of DEGs by quantitative real time RT-PCR

**Figure 5:**
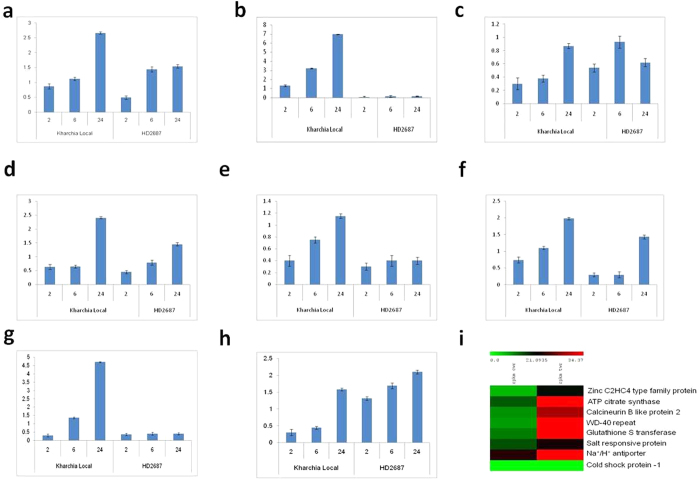
qRT-PCR validation of some selected genes. (**a**) zinc c3hc4 type family protein; (**b**) atp-citrate synthase; (**c**) calcineurin b-like protein 2; (**d**) wd40; (**e**) Na^+^/H^+^ antiporter (**f**) Salt responsive protein; (**g**) cold shock protein-1; (**h**) glutathione-s-transferase genes; (**i**) Heat map of expression profiles of some focused genes under salt stress conditions. The scale shows log2 fold change. A color version is available online.

To further validate the results from the 454 sequencing data, eight salt induced unigenes were selected randomly (based on their expression patterns) for qRT-PCR analysis of samples that were treated with 250 mM NaCl for 2, 12 and 24 hours and control in both salt tolerant (Kharchia Local) and susceptible (HD2687) wheat genotypes (Table 4, Supplementary Table S6). In the four stages of stress, the expression of the unigenes was up-regulated in both genotypes. However, expression level was higher in Kharchia Local (Fig. 5a–h). *Salt responsive protein* and *Na*^+^/*H*^+^ *antiporter* were not found to be significantly expressed in the transcriptome data analysis. To represent these unigenes under salt stress we created a heatmap of RPKM-normalized unigenes from 454 sequencing (Fig. 5i).

### Alternative splicing and Differential splicing analysis

Assembled transcripts are considered to be splice variants if they aligned to the same locus and transcribed from the same strand. Transcripts with low read support were filtered out as the transcripts with low read support are likely to be assembly artifacts. In total 621 genes were alternatively spliced, resulting in 6,375 transcripts (Table 5). In total, we found 50 differentially expressing splice variants (Supplementary Table S7).

### Identification of Simple Sequence Repeats (SSRs)

**Figure 6:**
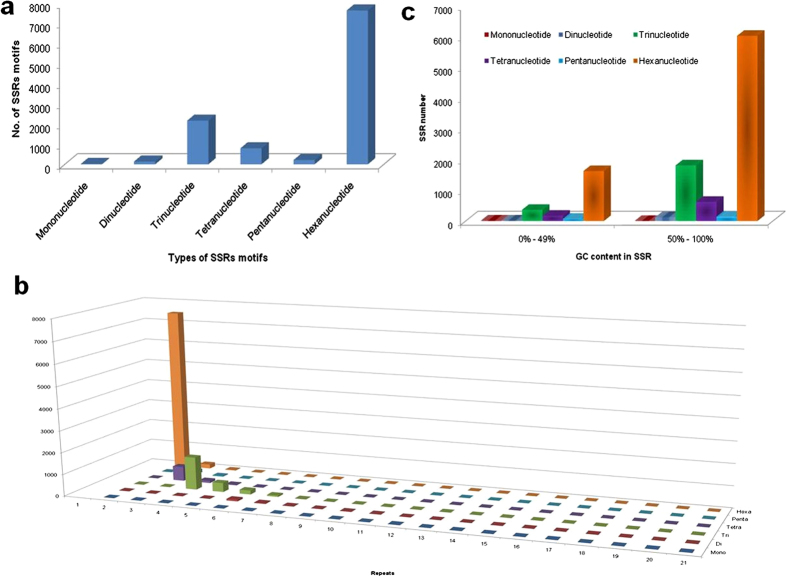
Distribution of SSRs. (**a**) Number of SSRs classified based on motif type; (**b**) Motif type and their frequency plotted as a function of the repeat number; (**c**) GC content in microsatellites among different nucleotide types found in the transcriptome of *T. aestivum*.

All the unigenes were used to mine potential SSRs to develop SSR markers. In total, 11,002 SSRs were identified by SSR Locator V.1 in 6,939 (38.74%) unigenes of *T. aestivum*. Out of these unigenes, 1,392 unigenes contained more than one SSR (Table 6). Hexa-nucleotide repeats (69.66%) were the most abundant SSRs in the transcriptome. The second major class was tri-nucleotide (19.77%) and the remaining repeat motifs were mononucleotide (0.16%), dinucleotide (1.24%), tetranucleotide (7.24%) and pentanucleotide (1.93%) (Fig. 6a). SSRs with two tandem repeats (67.71%) were the most common tandem repeat, followed by four, three, five and six tandem repeats (Fig. 6b). The dominant repeat motif in all the SSRs was CCG/CGG (269, 2.45%), followed by CGC/GCG (261, 2.37%), GCC/GGC (256, 2.4%), CTC/GAG (136, 1.24%) and GGA/TCC (121, 1.1%) (Supplementary Table S8). The analysis of GC content of microsatellites showed that most of the SSRs were rich in GC content, except for mono- nucleotide repeats (Fig. 6c). Focusing on tri- and hexa- nucleotide repeats, SSRs with 50–100% GC content were thrice as prevalent as those with only 0–50% GC content. SSRs can be used as functional markers in various genetic studies in the future.

## Discussion

With advances in functional genomics, proteomics and physiological studies, the molecular mechanisms of salinity stress tolerance are slowly being unraveled; however, more efforts are needed for deciphering the complexity of this stress factor in plants^37^. Next-generation sequencing technology provides a powerful tool for transcriptome analysis and the *de novo* assembly of transcript sequences is a rapid approach to identify expressed genes in non-model organisms^18^. The main aim of this study was to decipher the molecular mechanism of salt stress tolerance in wheat and to identify important genes and complex pathways that play a critical role in response to salt stress in *T. aestivum* at seedling stage. Salt stress at seedling stage is very crucial as most of the crop plants are sensitive at this developmental stage. We analyzed several physiological parameters under salt stress in wheat. A significant reduction in RWC, CHL, CAR and MSI and increase in proline and total soluble sugars content under salt stress was observed in the present study (Supplementary Fig. S1). To decode the molecular aspect of salt stress tolerance and investigate the relationship between physiological and molecular kinetics, we generated RNA-seq libraries from roots of Kharchia local variety of *T. aestivum* seedlings under normal growth conditions and in response to 24 h of salt stress. To our knowledge this is the first report of root transcriptome profiling of *T. aestivum* cv. Kharchia Local at seedling stage under salt stress.

We obtained over 453,882 raw reads leading to 17,911 predicted unigenes with N50 length of 648 bp and a total length of 10.39 Mb. Only 83.18% of these unigenes are represented in public database. Based on the annotation, some of the unigenes were closely related with the plant stress functions i.e. stress tolerance function (salt responsive protein, salt overly sensitive 1, salt tolerant like protein, salt induced protein, sodium hydrogen exchanger, sodium calcium exchanger, universal stress protein, stress protein, calcineurin B like protein, CBL-interacting protein kinase family), signal transduction (calmodulin, calcium calmodulin dependent protein kinase), energy production and conversion (ATP synthase beta subunit, ATP synthase subunit d, ATP synthase delta subunit, ATP binding protein, ATP citrate synthase, vacuolar ATP synthase subunit b, vacuolar ATPase b subunit, vacuolar type H^+^ ATPase, vacuolar proton ATPase b subunit) and inorganic ion transport (Na^+^/H^+^ antiporter, vacuolar proton-inorganic pyrophosphatase, transmembrane protein, plasma membrane H^+^-ATPase) (Supplementary Table S1). These putative functional unigenes identified in the present study, can provide leads for future investigations and valuable information for deciphering the putative function of novel genes. However, detailed studies are needed to understand their precise roles and functions.

Out of all the assembled unigenes, 12,199 unigenes were classified into 32 functional groups with 48.66% to biological process, 24.39% to molecular function and 26.95% to cellular component. This is indicative of the tissues undergoing extensive metabolic activities. Genes involved in some important biological processes such as response to stimulus (9.14%), single organism process (14.34%) and biological regulation (6.99%) were also identified. This was mostly in harmony with other analysis of DEG(s) under salt stress conditions (Fig. 2). These results suggest that *T. aestivum* may have unique genes that regulate the response to salt stress. Logically, genes encoding these functions may be more conserved across different species and are thus easier to annotate in the database.

Besides GO analysis, KEGG pathway mapping based on EC numbers for assignments was also carried out for the assembled sequence, which is an alternative approach to categorize gene functions with the emphasis on biochemical pathways. Orthology assignment and mapping of the contigs to the biological pathways were performed using KEGG automatic annotation server (KAAS). According to KEGG result, 6,143 unigenes were mapped onto a total of 310 predicted metabolic pathways (Table 2). Some stress signaling pathways represented in our study were “calcium signaling pathway”, “oxidative phosphorylation”, “fatty acid biosynthesis”, “flavonoid biosynthesis”, “ABC transporters” and “biosynthesis of other secondary metabolites”. At whole transcriptome level, KEGG pathway and COG analysis are very useful techniques for prediction of potential/putative genes and their functions. The predicted molecular pathways, together with COG analysis, are useful for further investigations of genes functions.

A total of 77 transcription factor family was identified in this study, out of which, WD-40 was most abundant followed by C2H2, MYB-HB-like, CCHC (Zn), PHD and bZIP. It has been already reported that WD-40 protein is expressed under salt stress in various plants such as BnSWD1 fromin *Brassica napus*^38^, SRWD in *Oryza sativa*^39^, SiWD40 in foxtail millet^40^ and WD40 repeat protein in *Medicago trunculata*^41^. Other TFs like C2H2 have been reported to play important role in biological processes and various abiotic stresses, involving cold, drought, oxidative and salt stress in rice^42^,^43^; cold and drought stress in soybean^44^ and osmotic, cold and mechanical stress in poplar^45^. This suggests that C2H2-ZFPs are likely to be associated with multiple physiological processes and stress responses in this salt tolerant land race of wheat also^46^. Over-expression of *OsMYB3R*-*2* MYB gene has been shown to increase tolerance to cold, drought and salt stress in *Arabidopsis*^47^. Over-expression of *OsMYB48-1* in rice improved tolerance to drought and salinity stresses^48^.

To identify significant gene expression changes associated with salt stress, differentially expressed genes (DEGs) were analyzed by comparing the CWR and TWR libraries. In total, 865 unigenes were categorized as up-regulated genes and 1,630 as down-regulated in TWR as compared to CWR (Fig. 4a). Of the 2,495 differentially expressed sequences, we predicted annotations for 2,329 unigenes against nr protein database (Fig. 4b). It is important to identify and understand the function and role of genes of unknown function which exhibited 10 fold change in expression level under salt stress. This will improve our understanding of the salt tolerance mechanism of this tolerant bread wheat cultivar Kharchia local and allow us to employ these strategies in improving tolerance to salt stress in exotic varieties of *T. aestivum*.

The salt stress tolerance in *T. aestivum* cv. Kharchia Local is capable of efficiently sequestering Na^+^ into the root cell vacuoles. This probably restricts the Na^+^ loading into the xylem. This process is implemented by transporting ions across tonoplast^49^. In the present study, V-ATPase (V-type proton-transporting ATPase; comp4743_c0_seq3) unigene was present at high levels in response to salt stress. V-ATPase is essential for making an electrochemical H^+^-gradient across tonoplasts to boost tonoplasts for efficient ion uptake into vacuoles through the tonoplast Na^+^/H^+^ antiporter (NHX)^37^. In our study, we observed higher transcript levels of V-ATPase, which provide more energy to form a stronger proton gradient to impel excessive cytoplasmic Na^+^ into vacuoles. These findings suggest that *T. aestivum* has tighter control over ion compartmentalization by adjusting the driving force of ion transport which is not observed in other varieties of bread wheat.

On exposure of abiotic stresses to plants, such as salinity, drought and low temperatures, plants exhibits an enhanced level of reactive oxygen species (ROS), superoxide radicals (O^−2^), hydrogen peroxide (H_2_O_2_), and hydroxyl radicals (^−^OH) that can disturb cellular homeostasis resulting in oxidative damage to cellular structures which leading to cell death^37^. The unigene associated with ROS scavenging-related gene “*glutathione S-transferases*” (GSTs; comp2115_c0_seq1) was also observed to be up-regulated in our study. Previously, over expression of GST has been reported to improve tolerance to abiotic stress in tobacco and soybean^50^,^51^. Peroxidases (PODs; comp103_c0_seq1), which are known to play an important role in H_2_O_2_ scavenging, was also over expressed in our study, which is in accordance with the results of salt-treated halophyte *Aeluropus littoralis*^52^. These results suggests that the ROS scavenging pathway enzymes play an important role in protecting cells from oxidative damage under salt stress.

The adjustment of energy metabolism under salt stress conditions is an important strategy to impart tolerance to salt stress^37^. We identified two unigenes coding for “*ATP citrate synthase*” (comp4786_c0_seq6) and “*atp synthase*” (comp4755_c0_seq2) which are related to energy metabolism. The expressions of these two unigenes were induced under salt stress. Another unigene homologous to “*cytochrome c oxidase*” (comp5253_c0_seq6), which is an electrostatically coupled energy transducer that contributes to the formation of ATP in aerobic life was found to be up-regulated in the present study^37^.

“*Cbl-interacting protein kinase* (CIPK; comp1008_c0_seq1)” and “*Calcineurin b like protein* (CBLs; comp8612_c0_seq1)” constitute a complex signaling network acting in diverse plant stress responses. Earlier studies in *Arabidopsis* and rice have shown the importance of Ca^2+^ signals in re-establishing cellular ion homeostasis, indicating the significance of the calcium sensor and signaling pathways involved in salt stress signal transduction^53^. The high salt stress tolerance of Kharchia local enabled us to identify a series of gene expression changes and snapshot of underlying salt-responsive genes. The enriched dominant GO terms that were identified during salt stress included “single organism process”, “metal ion binding”, “heme binding” which are in accordance with analysis of DEGs under salt stress conditions in other plans^37^. This suggests that different genes usually cooperate with each other to exercise their molecular function.

**Figure 7:**
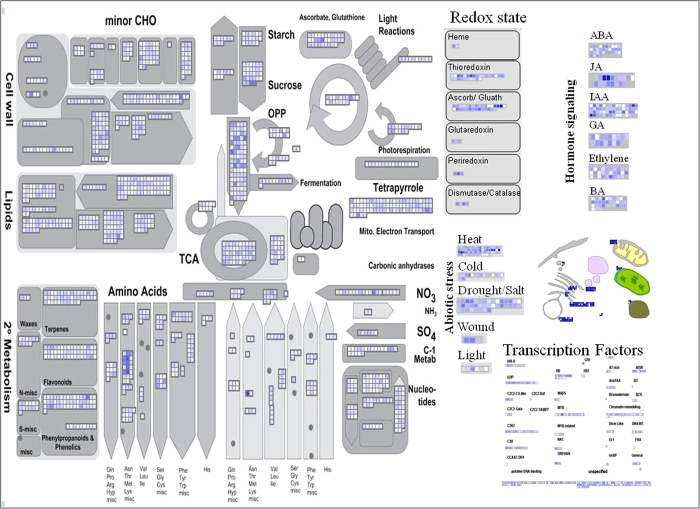
MapMan depicting gene regulation in functional categories associated with different pathways in *T. aestivum*.

To further explore, we mapped DEGs on MapMan tool (Fig. 7). Among the TF family, zinc finger, bHLH, bZIP transcription factors were up-regulated in response to salt stress. Genes mediating ROS detoxification including *peroxiredoxin* (*POD*), *thioredoxin* (*Trx*) and *glutathione peroxidase* (*GPX*) have also shown up-regulation along with several key abiotic stress responsive genes involving sulphur assimilation (*APR*, *ATPS* and *AKN*). Sulphur assimilation involves activation of sulfate to adenosine 5′-phosphosulfate (APS) by ATP sulfurylase (ATPS) which is phosphorylated by APS kinase (AKN). Subsequently, APS is reduced to sulfite by APS reductase (APR). Sulfite is reduced to sulfide and incorporated into cysteine which is a precursor of glutathione (GSH). We observed significant increase in the transcript levels of *APR* gene in response to salt stress in our study (Fig. 7).

Generation of splice variants is an important mechanism for regulating gene expression and for increasing transcriptome plasticity and proteome diversity in eukaryotes. Presence of splice variants plays important role in various physiological processes in plants. This includes response to different abiotic biotic and stresses. There are very few studies where alternative splicing in response to stress have been studied. Large-scale or transcriptome based studies of splice variants under salt stress conditions are still relatively less. In the present study, some of the unigenes containing splice variants were showing homology with (based on the annotation with NCBI Plant RefSeq database): drought and cold related proteins, glutathione-s-transferase, β-glucosidase, salt responsive protein, Na^+^/H^+^ antiporter, zinc c3hc4 type family protein, atp-citrate synthase, wd-40 repeat, calcineurin b-like protein 2, sucrose phosphate synthase (Supplementary Table S7). These results suggest that these genes played important role in imparting tolerance to salt stress. Out of 50 differentially expressing splice variants, some unigenes showed homology with cell wall associated hydrolase, udp-glucuronic acid decarboxylase 1, lipoxygenase 2, phenylalanine ammonia-lyase, and sucrose synthase. There were more than two splice variants of these unigenes in the present transcriptome of Kharchia Local. Interestingly all of these unigenes are known to play important role in salinity stress tolerance in plants^54^,^55^,^56^,^57^,^58^. Ding *et al*.^59^ reported that in *Arabidopsis* there is an increase in alternate splicing frequency under salt-stress conditions^59^. Alternate splicing seems to represent an additional level of gene regulation in response to salt stress, however, further comprehensive analysis is needed in this direction. It can reveal novel insights into the role of alternate splicing in response to various abiotic stresses in plants.

Molecular markers play an important role in plant biology. These are commonly used for the analysis of plant genomes and identification of the association between genomic variation and heritable traits. In our study a total of 11,002 SSRs were identified. These were mostly hexa-nucleotide repeats and tri-nucleotide (Fig. 6a,b). To make these SSR markers useful, we employed Primer 3 to design primer pairs for each SSR. In total, 8,181 primer pairs for 11,002 (74.35%) SSRs were designed from the microsatellites with sufficient flanking sequences (Supplementary Table S9). Further validation in relation phenotypic trait is beyond the scope of this report and will be discussed in another manuscript.

In conclusion, the comparative root transcriptome of *T. aestivum* cv. Kharchia Local showed multiple genes and pathways involvement to gain tolerance against salt stress. Our finding enriches the presently available genomic resources and will give an impetus to research on improving the salt tolerance in important wheat varieties. The data generated in our study can be a valuable resource and support genome analysis, besides aiding in developing expression analysis platforms, identification of molecular marker identification and initiate functional and comparative genomic studies. Our comprehensive study represents a valuable contribution towards strategies for improving the salt tolerance of crop plants.

## Methods

### Plant Growth and Stress Treatment

Seeds of Kharchia Local (*T. aestivum*, 2n = 42, AABBDD) were collected from ICAR-Central Soil Salinity Research Institute (CSSRI), Karnal, India. Seedlings were raised in hydroponics in Hoagland solution with 16/8 h light/dark at 24/18 °C, irradiance of 150 μmol m^−2^s^−1^ and air humidity of 60% at National Phytotron Facility, IARI Campus, New Delhi. After 10 days 250 mM salt (NaCl) stress was imposed for 24 h. The root samples were taken from both control and treated seedlings and processed for RNA isolation.

### Total RNA isolation and mRNA extraction

Total RNA was isolated from both the samples (control, treated) using RaFlex^™^ Total RNA Isolation Kit (GeNei^™^, Bangalore, India) as per the manufacturer’s protocol. Contaminating genomic DNA was removed by DNase I (Qiagen, Hilden, Germany) treatment. The quality of the total RNA was checked by running the samples on 1.2% formaldehyde agarose gel electrophoresis under denaturing conditions.

mRNA was isolated from total RNA using Oligotex^®^ mRNA Midi Kit (Qiagen, Hilden, Germany) as per the manufacturer’s protocol. The integrity of mRNA was checked on a 1.2% agarose gel containing formaldehyde and its quantity was measured using Nano Drop 2000 spectrophotometer (Thermo Scientific, Wilmington, DE, USA). Equal quantities of high-quality mRNA from biological replicates were pooled together and were used for transcriptome study.

### cDNA Library construction and 454 sequencing

cDNA was synthesized from the pooled RNA samples of CWR and TWR using cDNA Synthesis System (Roche, Basel, Switzerland) as per the manufacturer’s protocol using hexamer primers. The cDNA samples were sheared by nebulization to produce random fragments of approximately 300–800 bp in length. The nebulized cDNAs were recovered using QIAquick^®^ PCR Purification Kit (Qiagen, Hilden, Germany). Rapid Library Molecular Identifiers (RLMIDs) were ligated to the fragmented purified cDNA samples according to the standard protocol. Each MID contains a barcode sequence that is used to discriminate samples from different libraries. Library quantification was done using TBS 380 Fluorometer. The Relative Fluorescence unit of each dilution was recorded and checked against RL standard curve and the sample concentration was calculated. Agilent Bioanalyzer (Agilent Technologies, Palo Alto, CA, USA) High Sensitivity DNA chip was used to check quality of the libraries.

The quality passed samples were pooled and amplified by emulsion PCR for sequencing. The sequencing of the libraries was performed on 454 – GS FLX Titanium sequencer (454 Life Sciences, Roche, Basel, Switzerland).

### Sequence data analysis and assembly

The raw reads were first pre-processed by eliminating adaptor sequences using customized Perl scripts. All the sequences smaller than 60 bases were eliminated based on the assumption that small reads might represent sequencing artifacts. At the same time Q20, Q30, GC content and sequence duplication level of clean data were calculated. Finally, high quality reads were assembled into unique putative transcripts, termed as unigenes (including contigs and singletons), using Trinity ESTs assembler. The assembly was performed using the default parameters.

### Functional annotation, GO classification and metabolic pathway analysis of unigenes

Blast homology searches and sequence annotations were carried out using Blast2GO tool v.3.1.3 (http://www.blast2go.org)^32^,^33^,^34^. The assembled sequences were compared against the NCBI non-redundant (nr) protein database via Blast X, using an E-value cut-off of 1.0 E^−3^. For gene ontology mapping, the program extracts the GO terms associated with homologies (GO; http://www.geneontology.org). These GO terms are assigned to query sequences, producing a broad overview of groups of genes cataloged in the transcriptome for each of three GO categories. The data presented herein represent a GO analysis at level 2, illustrating general functional categories. GO enrichment analysis was also done. This enrichment analysis was performed to evaluate the enrichment of various GO categories for the unigenes having 2 fold or above expression values in both the samples. The unigene sequences were also aligned to the COG database to predict and classify proteins. KEGG pathways were assigned to the assembled sequences using the online KEGG Automatic Annotation Server (KAAS)^60^, http://www.genome.jp/kegg/kaas. The single-directional best hit (SBH) method was used to obtain KEGG analysis. For identification of transcription factor in Kharchia Local transcriptome, all the assembled unigenes were analyzed against the PlantTFcat online tool (http://plantgrn.noble.org/PlantTFcat). GC content of the sequences was measured using Emboss GeeCee tool (http://emboss.bioinformatics.nl/cgi-bin/emboss/geecee).

### Expression analysis

The RPKM method was used to calculate the unigene expression in this study^61^. The clean reads of each library were mapped to the sequences of each unigene. The significance of difference in gene expression between the control and salt treated plants was determined by using DEGseq, an R package^62^. False discovery rate (FDR) was applied to identify the threshold of the P-value in multiple tests^63^. When FDR was less than 0.05 and log ratio greater than 1 (two-fold change) between the samples the unigenes were considered as differentially expressed.

### Alternative splicing Landscape and Differential splicing analysis

To analyze the various types of Alternative splicing (AS) raw reads of both the samples (CWR and TWR) were mapped to wheat reference genome released by IWGSC (http://plants.ensembl.org/Triticum_aestivum), using TOPHAT software using default parameters, which utilizes computational methods to detect variants and splicing isoforms in short reads through merging and filtering position lists from a genomic index. For differential splicing analysis, we used Cuffdiff, which categorizes individual transcripts based on their transcription start site, thus grouping together all the transcripts with a common pre-mRNA molecule^64^,^65^. Cuffdiff then estimates significant differences in expression of each transcript in a group relative to one another using the Jensen-Shannon divergence metric across the different samples. Differential expression calls were made using the DESeq package.

### Physiological analysis

Relative water content, chlorophyll and carotenoid content, membrane stability index, proline content and total soluble sugars were measured in control and salt treated plants at seedling stage as mentioned by Sairam *et al.,* (2012)^66^. All the experiments were performed with three biological and three technical replicates.

### Validation of genes using qRT-PCR

Seeds of Kharchia Local (salt tolerant) and HD2687 (salt susceptible) seeds were grown in hydroponics as mentioned above. The 10 days old seedlings were subjected to 250 mM NaCl stress. The root samples of salt treated samples at different time points (2, 12 and 24 h) and control were taken, and total RNA was isolated using the same method mentioned above. The salt-induced expression of unigenes with potential roles in the salt stress response was chosen for validation using qRT-PCR. Three independent biological samples of each were used in the analysis. cDNA was synthesized from 2 μg of total RNA using Superscript^®^ III First strand cDNA Synthesis system (Invitrogen, USA) as per the manufacturer’s protocol. *Actin* is reported to exhibit most stable expression in different developmental stages and stresses, was used as reference for gene expression normalization. The gene specific primers are listed in Supplementary Table S8. qRT-PCR was performed using an Agilent Mx3000P^™^ PCR platform and KAPA SYBR^®^ FAST qPCR kit Master Mix (2X) Universal (Kapa Biosystems, Woburn, MA) as per manufacturer’s instructions. The relative expression levels of the selected unigenes normalized to the expression level of actin were calculated from cycle threshold values using the 2^−ΔΔCt^ method. This experiment was carried out in three independent biological replicate and three technical replicate of each biological replicate.

## Additional Information

**How to cite this article**: Goyal, E. *et al*. Transcriptome profiling of the salt-stress response in *Triticum aestivum* cv. Kharchia Local. *Sci. Rep.* **6**, 27752; doi: 10.1038/srep27752 (2016).

## Supplementary Material

Supplementary Information

Supplementary Table S1

Supplementary Table S2

Supplementary Table S3

Supplementary Table S4

Supplementary Table S5

Supplementary Table S6

Supplementary Table S7

Supplementary Table S8

Supplementary Table S9

## Figures and Tables

**Table 1:** Summary of 454 transcriptome sequencing and assembly for *T. aestivum*.

**Item**	**Library**	**Number**	**Total Bases (Mb)**
Raw read	CWR	106,649	57.4
	TWR	347,233	187.4
Clean read	CWR	98,245	52.1
	TWR	268,777	170.5
Average Length (bp)	CWR	534	
	TWR	532	
Unigenes			
No. of Unigenes (n)		17,911	–
Average Length (bp)		579	–
Maximum Length (bp)		3,602	–
Minimum Length (bp)		206	–

**Table 2:** KEGG classification of *T. aestivum* unigenes.

**KEGG categories**	**No. of unigenes**	**KEGG categories**	**No. of unigenes**
**Metabolism**	**1,390**	**Environmental Information Processing**	**218**
Carbohydrate Metabolism	1147	Signal Transduction	931
Energy Metabolism	473	Membrane Transport	42
Lipid Metabolism	485	Signaling & Interaction Molecules	7
Nucleotide Metabolism	205	**Organismal System**	**349**
Amino Acid Metabolism	761	Immune System	270
Metabolism of other Amino Acid	248	Endocrine System	386
Glycan Biosynthesis & Metabolism	137	Circulatory System	98
Metabolism of Cofactors and Vitamins	145	Digestive System	102
Metabolism of Terepenoids & Polyketides	141	Excretory System	89
Biosynthesis of other Secondary Metabolites	274	Nervous System	311
Xenobiotics Degradation & Metabolism	163	Sensory System	61
**Genetic Information Processing**	**633**	Development	43
Transcription	243	Environmental Adaption	216
Translation	657	**Human Disease**	**668**
Folding, Sorting & Degradation	685	Cancer	589
Replication & Repair	169	Immune Disease	59
**Cellular Process**	**289**	Neurodegenerative Disease	424
Transport & Catabolism	465	Substance Dependence	76
Cell Growth & Death	408	Circulatory Disease	120
Cell Motility	74	Endocrine & metabolic Disease	142
Cell Communication	185	Infectious Disease	503
		**Total**	**6,143**

**Table 3:** GO enrichment analysis of differentially expressed genes.

**GO-ID**	**Term**	**Category**	**No. of differentially expressed genes in subgroup**	**No. of unigenes in subgroup**	**P-value**	**FDR**
Up-regulated in CWR						
**Total**			**842**	**12,199**		
GO:0005506	iron ion binding	F	3	2	1.83E-03	6.73E-01
GO:0009653	anatomical structure morphogenesis	P	50	557	8.75E-03	6.58E-01
GO:0042623	ATPase activity, coupled	F	2	1	9.86E-03	6.58E-01
GO:0044699	single-organism process	P	206	2865	1.18E-02	6.58E-01
GO:0046914	transition metal ion binding	F	23	217	1.76E-02	6.58E-01
GO:0016705	oxidoreductase activity, acting on paired donors, with incorporation or reduction of molecular oxygen	F	2	2	1.90E-02	6.58E-01
GO:0016049	cell growth	P	25	257	2.51E-02	6.58E-01
GO:0016773	phosphotransferase activity, alcohol group as acceptor	F	57	694	2.51E-02	6.58E-01
GO:0004672	protein kinase activity	F	57	694	2.51E-02	6.58E-01
Up-regulated in TWR						
**Total**			**239**	**12,199**		
GO:0007017	microtubule-based process	P	2	0	1.07E-02	1.00E+00
GO:0003682	chromatin binding	F	25	127	1.31E-02	1.00E+00
GO:0003774	motor activity	F	16	77	2.85E-02	1.00E+00
GO:0008092	cytoskeletal protein binding	F	24	136	4.02E-02	1.00E+00
GO:0016462	pyrophosphatase activity	F	16	81	4.03E-02	1.00E+00
GO:0017111	nucleoside-triphosphatase activity	F	16	81	4.03E-02	1.00E+00
GO:0016818	hydrolase activity, acting on acid anhydrides, in phosphorus-containing anhydrides	F	16	81	4.03E-02	1.00E+00
GO:0006996	organelle organization	P	108	788	4.87E-02	1.00E+00

**Table 4:** ESTs used for validation of gene expression profile of *T. aestivum* transcriptome data.

**Unigene_ID**	**Length (bp)**	**CWR_RPKM**	**TWR_RPKM**	**Fold Change**	**Gene description**	**Accession No. of homolog (Organism)**	**E-Value**
comp3309_c0_seq1	490	–	–	–	Actin 1	EMS62134 (*T.urartu*)	2.34E-114
comp9193_c0_seq1	1028	14.84	22.183	1.495	Salt responsive protein	ABG75754.1 (*T. aestivum*)	1.81E-79
comp511_c0_seq1	1327	22.992	34.37	1.495	Na^+^/H^+^ antiporter	AAK76737.1 (*T. aestivum*)	4.01E-134
comp7516_c0_seq1	1140	6.691	20.004	2.99	Zinc C3HC4 type family protein	BAJ89228.1 (*H. vulgare*)	5.98E-143
comp4786_c0_seq6	541	14.099	56.203	3.986	ATP-citrate synthase	XP_003579058.1 (*B. distachyon*)	1.34E-69
comp8612_c0_seq1	776	9.829	29.387	2.99	Calcineurin b-like protein 2	EMS57734.1 (*T. urartu*)	3.39E-119
comp6597_c0_seq1	851	8.963	35.73	3.986	WD-40 repeat	EMT05171.1 (*A. tauschii*)	1.77E-143
comp4247_c0_seq1	209	0	0	1	Cold shock protein-1	BAD08701.1 (*T. aestivum*)	1.09E-28
comp2115_c0_seq1	665	11.47	80.016	6.976	Glutathione S-Transferase	XP_003575165.1 (*B. distachyon*)	1.03E-118

**Table 5:** Statistics of Splice variants in *T. aestivum* transcriptome.

**Item**	**Number**
No. of Unigenes (n)	17,911
No. of unigenes containing Splice Isoforms (n)	6,375
No. of unigenes containing g Splice variants (n)	621
Differentially expressing splice variants (n)	50
Max Length (bp)	3,602
Min Length (bp)	201
Average Length (bp)	579.8
N50 (bp)	648

**Table 6:** Statistics of SSRs in the *T. aestivum* transcriptome.

**Items**	**Numbers**
Total length of sequence (bp)	10,386,170
Total no. of SSRs	11,002
No. of SSR containing sequences	6,939 (38.74%)
No. of sequences containing more than one SSR	1,392
No. Of SSR present in compound formation	1,389
Frequency of SSR Kb/one SSR	0.94
No. of mono-nucleotide SSR	18 (0.16%)
No. of di-nucleotide SSR	137 (1.25%)
No. of tri-nucleotide SSR	2,175 (19.77%)
No. of tetra-nucleotide SSR	796 (7.24%)
No. of penta-nucleotide SSR	212 (1.93%)
No. of hexa-nucleotide SSR	7,664 (69.66%)
