# Supplementary material for: Transcriptome profiling of the salt-stress response in Triticum aestivum cv. Kharchia Local
Source: Sci Rep. 2016 Jun 13;6:27752. doi: 10.1038/srep27752 (PMC4904219; doi:10.1038/srep27752)
Supplement: Supplementary Information [file srep27752-s1.doc]

**Title: Transcriptome profiling of the salt-stress response in *Triticum aestivum* cv. Kharchia Local**

***Etika Goyal^1, 2^, Singh K. Amit^2^, Ravi S. Singh^2^, Ajay K. Mahato^2^, Suresh Chand^1,3^ and Kumar Kanika^2*^***

[1] Banasthali University, Banasthali, Rajasthan

[2] Biotechnology and Climate Change Laboratory, ICAR-NRC on Plant Biotechnology, New Delhi

[3] Devi Ahilya University, Indore

*Corresponding Author**

Dr. Kanika

Senior Scientist

Biotechnology and Climate Change Laboratory, Lal Bahadur Shastri Building, ICAR-National Research Centre on Plant Biotechnology, Indian Agricultural Research Institute Campus, New Delhi – 110012

E-mail: kumarkanika@rediffmail.com; goyal.etika@gmail.com; selfamitbt@gmail.com.


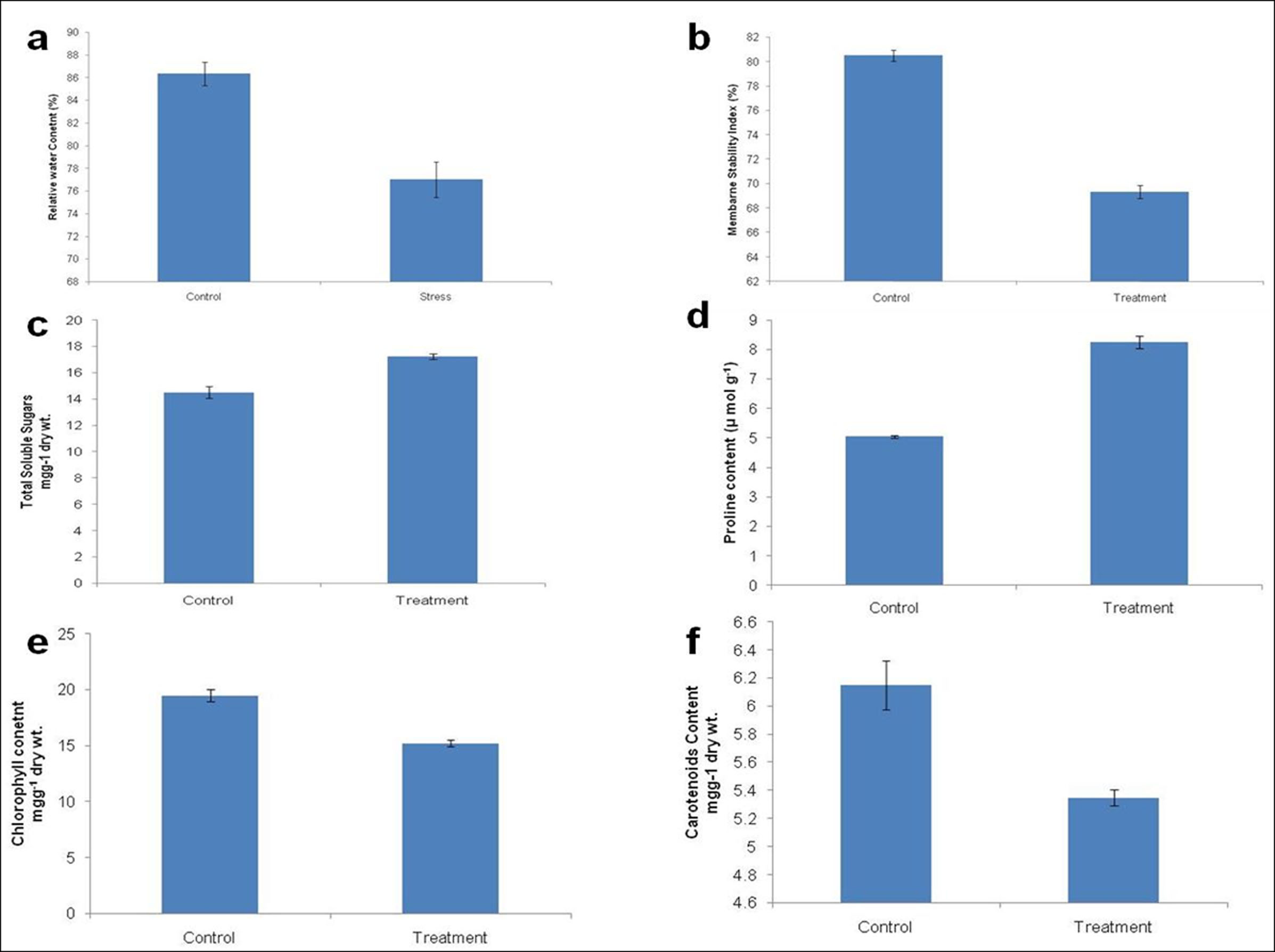


**Supplementary Fig. S1: Physiological analysis of *T. aestivum*.** (a) RWC; (b) CAR; (c) CHL; (d) MSI; (e) Proline; (f) TSS.

**
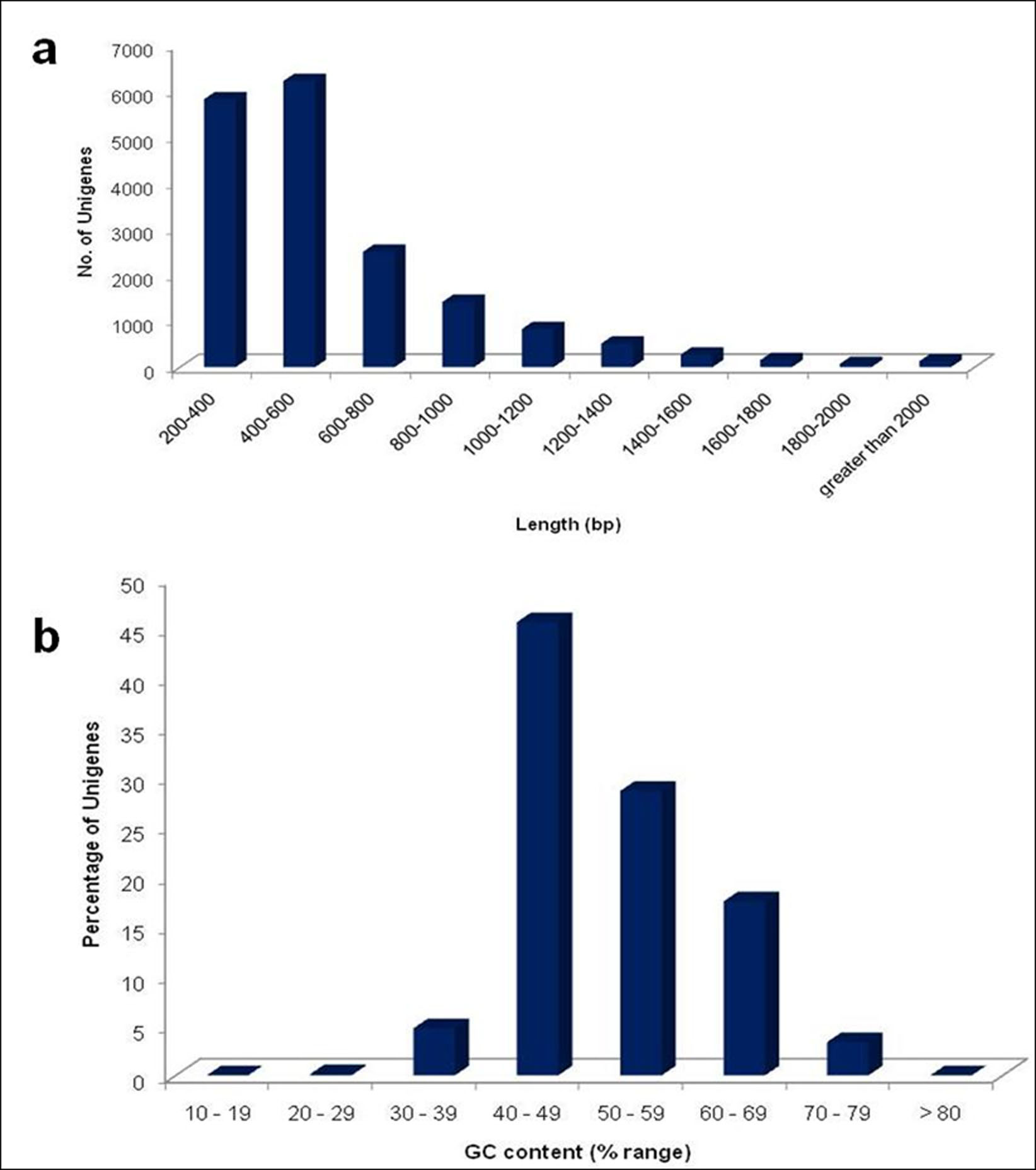
**

**Supplementary Fig. S2:** **Overview of *T. aestivum* transcriptome assembly.** (a) Size distribution of unigenes. Y-axis: number of unigenes; X-axis: Size in bp. (b) GC content of the unigenes.
